# Diseases of Certain Glands

**Published:** 1905-03-04

**Authors:** 


### DISEASES OF CERTAIN GLANDS.

**Diseases of the Thyroid.**—Hirschl[1] showed at Vienna a case which gave the symptoms of both Graves' disease and of Addison's disease. The patient was a brewer, aged 36 years. For a year he had lost flesh, very rapidly lately (70 lb. in four months). He then presented the following groups of symptoms—on the one hand, loss of motor power, bronzing of the skin, but with no pigmentation of the mouth, excitability, and forgetfulness ; on the other, goître, exophthalmos with the three eye symptoms, palpitation, and tremor. There was a family history of, but no physical sign pointing to, tuberculosis. Pindes[2] brings forward clinical and experimental evidence to show that the main thyroid gland is related to cachexia, while the parathyroids are related to tetany. Thus total thyroid removal, or removal of the parathyroids alone, leads to tetany, while removal of the main thyroid gland, with retention of the parathyroids produces cachexia but not tetany. The parathyroids develop from the third and fourth branchial clefts, and are found connected with the capsules of the lateral lobes. Hence removal of both lobes of the thyroid is more likely to be followed by tetany than removal of the isthmus. Shattuck[3] does not consider that there is sufficient evidence to connect Graves' disease with anomalies or injury of the parathyroids. In treating this disease, he lays great stress on the diet and general régime. An absence of highly nitrogenous food, with an abundance of carbohydrates and fat are indicated. Constipation or diarrhœa, which so often occur, should be treated with calomel or bismuth. The natural tolerance of these patients for quinine is remarkable, and the neutral bromide of quinine is spoken of as being of more value than any other drug in checking the gastro-intestinal symptoms. Mayo[4] discusses the prospects and limitations of surgical treatment in exophthalmic goître. He admits that many cases recover under the treatment of the "internist," but suggests that similarly many cases recover with no treatment at all. Every case in which he considered operation justified had had a long course of diet and medicine previously without avail. The thyroid and supra-renal glands produce internal secretion of an exactly opposite physiological effect; that of the thyroid tending to dilate the peripheral vessels and lower the blood pressure, whilst that of the supra-renals increases the blood pressure, contracts the capillaries and thus greatly lessens the peripheral circulation. In normal conditions a state of equilibrium is maintained between these two conditions. If either is absent or diminished the other produces an unbalanced effect. Thus the cretinism associated with absence of thyroid results from the uncontrolled action of the supra-renals contracting all the peripheral circulation, and thus preventing growth. The following operative measures have been attempted. (1) Exothyropexy, or the permanent exposure of the gland by lifting its lobes external to the skin. This is not performed now. (2) Ligation of the thyroid arteries. (3) Operations upon the cervical sympathetic ganglia. (4) Thyroidectomy. [The action of the $x$-rays are extremely beneficial, either as a curative agent or as a preliminary to operation. In 10 cases where $x$-rays were tried, all were benefited by a lessening of the tachycardia, irritability and exophthalmos, and the gland itself became smaller, harder and better defined. A review is given of 40 cases operated on by the author; of these six died. The anæsthetics used were cocaine, chloroform, or ether, but there seemed no advantage in using the first. The half-gland sometimes with the isthmus is removed. Some degree of thyroidism is commonly present for a few days after the operation. This is reduced to a minimum by draining the wound freely and giving supra-renal extract combined with morphia or atropine. All cases which survived the operation showed marked improvement, of these about half recovered from the tachycardia, nervousness and tremors at once. The exophthalmos is the last symptom to disappear. Cases in which the pulse is 130 to 160 with fluctuations in tension and rapidity, and in which there is anæmia and swelling of the feet, are unfit for operation, but often improve under belladonna and the $x$ ray treatment sufficently for operation. Posey[5] describes a case of Graves' disease in a woman of 43, in which there was paralysis of the sixth and part of the third nerve. Paralyses of other cranial nerves have been reported in the same connection, but necropsies have always given a negative result, suggesting that the paralysis is of a functional nature. Thompson,[6] after reviewing recent theories regarding the functions of the thyroid, suggests that the thyroid secretion may be of an antitoxic nature. It is suggested that the toxin of Graves' disease is of gastro-intestinal origin, and that it acts upon the nervous system, and that the best treatment of the condition is directed to modifying the production of this toxin by diet rather than by any symptomatic or surgical procedures. Thompson believes that the thyroid is intimately associated with the nutrition of connective tissue, and he mentions the possibility of relieving the symptoms of inoperable gliomata by the administration of thyroid extract, and he mentions one case in which this was successfully accomplished.

The Supra-renal Glands.—Minervini[7] describes the development and discusses the functions of these structures. He finds they appear in the hen on the fourth day of incubation above the Wolffian body and germinal epithelium. They are probably a part of the pronephros. In the human fœtus they are always larger than the kidneys, and show no differentiation into central and cortical zones. He finds no nerve-cells in fœtal life, but describes some vascular glomeruli, which atrophy later. As regards function he states that, besides containing two distinct substances which raise the blood-pressure by causing arterial contraction, they neutralise various poisons of endogenous origin, especially those proceeding from the disintegration of muscle.

[1] Med. Rec., July, 1904. [2] Ibid., July, 1904. [3] Ibid., July, 1904. [4] Ibid., Nov. 5, 1904. [5] Lancet, Aug. 13, 1904. [6] Med. Rec., Nov. 19, 1904. [7] Lancet, Jan. 21, 1905.

